# *N*-myristoyltransferase proteins in breast cancer: prognostic relevance and validation as a new drug target

**DOI:** 10.1007/s10549-020-06037-y

**Published:** 2021-01-04

**Authors:** John R. Mackey, Justine Lai, Utkarsh Chauhan, Erwan Beauchamp, Wei-Feng Dong, Darryl Glubrecht, Yie-Wei Sim, Sunita Ghosh, Gilbert Bigras, Raymond Lai, Luc G. Berthiaume

**Affiliations:** 1 Pacylex Pharmaceuticals, Inc., Edmonton, AB T5J 4P6 Canada; 2 Department of Oncology, Faculty of Medicine and Dentistry, University of Alberta, Edmonton, AB T6G 2H7 Canada; 3 Department of Laboratory Medicine and Pathology, Faculty of Medicine and Dentistry, University of Alberta, Edmonton, AB T6G 2H7 Canada; 4 Faculty of Medicine and Dentistry, University of Alberta, Edmonton, AB T6G 2H7 Canada; 5 Department of Cell Biology, Faculty of Medicine and Dentistry, University of Alberta, Edmonton, AB T6G 2H7 Canada

**Keywords:** Myristoylation, *N*-myristoyltransferase, Immunohistochemistry, Breast cancer, Prognosis, PCLX-001

## Abstract

**Purpose:**

*N*-myristoyltransferases 1 and 2 (NMT1 and NMT2) catalyze the addition of 14-carbon fatty acids to the N-terminus of proteins. Myristoylation regulates numerous membrane-bound signal transduction pathways important in cancer biology and the pan-NMT inhibitor PCLX-001 is approaching clinical development as a cancer therapy. The tissue distribution, relative abundances, and prognostic value of the two human NMTs remain poorly understood.

**Methods:**

We generated and validated mutually exclusive monoclonal antibodies (mAbs) specific to human NMT1 and NMT2. These mAbs were used to perform immunohistochemical analysis of the abundance and distribution of NMT1 and NMT2 in normal breast epithelial samples and a large cohort of primary breast adenocarcinomas from the BCIRG001 clinical trial (*n* = 706).

**Results:**

NMT1 protein was readily quantified in normal and most transformed breast epithelial tissue and was associated with higher overall histologic grade, higher Ki67, and lower hormone receptor expression. While NMT2 protein was readily detected in normal breast epithelial tissue, it was undetectable in the majority of breast cancers. Detectable NMT2 protein correlated with significantly poorer overall survival (hazard ratio 1.36; *P* = 0.029) and worse biological features including younger age, higher histologic grade, lower hormone receptor expression, higher Ki67, and p53 positivity. Treatment of cultured breast cancer cells with PCLX-001 reduced cell viability in vitro. Daily oral administration of PCLX-001 to immunodeficient mice bearing human MDA-MB-231 breast cancer xenografts produced significant dose-dependent tumor growth inhibition in vivo.

**Conclusions:**

These results support further evaluation of NMT immunohistochemistry for patient selection and clinical trials of NMT inhibition in breast cancer patients.

**Supplementary Information:**

The online version of this article (10.1007/s10549-020-06037-y) contains supplementary material, which is available to authorized users.

## Introduction

Fatty acylation is the covalent modification of proteins by fatty acid moieties. The attachment of a 14-carbon myristoyl group, called myristoylation, is one such example and it plays a critical role in the targeting of proteins to the endomembrane and plasma membrane systems, cell viability signaling, as well as protein–protein interactions [1, 2]. Two *N*-myristoyltransferases (NMTs), NMT1 and NMT2, catalyze the co- and post-translational myristoylation of approximately 2% of proteins encoded by the human genome; 364 different proteins representing 589 proteoforms have recently been identified [3]. Key myristoylated proteins in human cells include those that regulate cell growth and apoptosis such as c-Src, c-Abl, G_α_ subunits, and caspase-truncated (ct-) Bid and ct-PAK2 [1]. Although NMT1 and NMT2 share similar protein structures, biochemical and kinetic studies indicate that they have different substrate affinities and are not functionally redundant [4, 5]. NMT protein levels have also been reported to be increased in some cancers and thus, NMTs have been proposed to be potential therapeutic targets [6, 7].

Despite being of key importance in cellular signalling and function, we have limited understanding of NMT abundance and the distribution of these proteins in human tissues. Genome-wide transcriptome profiles of human tissues have inherent problems due to the unstable nature of RNAs, the highly variable relationships between RNA and protein abundance, and the fact that most tissues are comprised of different types of cells, each with their own unique expression pattern. Immunohistochemistry (IHC) analysis of NMT1/NMT2 cell-specific protein levels and distribution has, to date, been relatively rudimentary. This is primarily due to the use of polyclonal antibodies raised against NMT1 with unknown cross reactivity with NMT2 [8, 9]. The first report of NMT immunohistochemistry in human tissues comes from a 1997 paper [8] that used polyclonal antibodies resulting from immunization with the peptide NENYVEDDDNMFRFD. It is noteworthy that this sequence is identical in NMT1 (amino acids 177–191) and NMT2 (amino acids 179–193). Using this polyclonal antibody, Shrivastav et al. concluded there was increased NMT1 protein in oral squamous cell carcinoma compared to normal oral mucosa [9], although it is not possible to distinguish the relative contribution of NMT1 and NMT2 given the identity of the immunogen peptide sequence used.

To clarify the physiologic distribution, range of NMT protein levels in malignancy, and evaluate their potential prognostic value, we generated and validated selective, high-specificity, mutually exclusive monoclonal antibodies (mAb) against NMT1 and NMT2 suitable for analysis of paraffin-embedded formalin fixed tissues. Using these anti-NMT1 and anti-NMT2 mAb preparations, we initiated an immunohistochemical tissue survey of NMT1 and NMT2 protein levels in a large cohort of normal and breast cancer tissue samples and sought relationships among NMT protein levels, clinical outcomes, and pathologic features of the breast tumors. We found that NMT1 protein levels correlated with poor prognosis in general and that NMT2 protein could not be detected in the majority of breast cancer samples and when it was detected it was linked with a poorer prognosis. While NMTs have been proposed to be potential anti-cancer targets, herein we demonstrate the potent pan-NMT inhibitor PCLX-001 [10–12] can efficiently target breast cancer by reducing the viability of numerous breast cancer cell lines in vitro and cause disease regression in vivo in an established murine xenograft breast cancer model.

## Materials and methods

### Cell lines, cell culture, and western blotting

NMT1 and NMT2 normal IM9 was purchased from ATCC while NMT2-deficient BL2 cell line was a kind gift of Dr. Robert Ingham of University of Alberta. The identity of both cell lines was confirmed at The Genetic Analysis Facility (Toronto, ON, Canada; www.tcag.ca). The sensitivity of Au-565, BT-20, BT-549, DU4475, Hs 578 T, and MCF7 breast cancer cell lines to PCLX-001 was performed by the Netherlands Translational Research Center B.V. Oncolines™ (www.oncolines.com). MDA-MB-231 breast cancer cell line for xenograft experiments were maintained by Pharmaron laboratory facilities (Beijing, China). All cell lines were maintained in RPMI or DMEM medium supplemented with 5–10% fetal bovine serum, 100 U/ml penicillin, 0.1 mg/ml streptomycin, 1 mM sodium pyruvate, and 2 mM l-glutamine. Tissue culture growth medium and supplements were from Gibco-Life Technologies (Burlington, ON, Canada). All cell lines were maintained at 37 °C and 5% CO_2_ in a humidified incubator and routinely checked for contaminating mycoplasma.

Cells were harvested and processed for western blotting as described in the supplementary methods. Protein concentrations were determined by BCA assay (Thermo Scientific) according to manufacturer’s instructions and unless otherwise, 40 μg of total protein per lane was loaded on 12.5% acrylamide gels. After electrophoresis, gels are transferred onto 0.2 μM nitrocellulose membrane (Bio-Rad), blocked and probed with sera and antibodies as described. Peroxidase activity was detected following the procedure provided for the ECL Prime Western Blotting Detection Reagent (GE Healthcare, PA, USA).

### Monoclonal antibody production and validation

Human mouse monoclonal anti-human NMT1 and NMT2 antibodies were produced using hybridoma technology [13] by GenScript (Piscataway, NJ, USA) using human NMT1 specific QDQPVKMNSLPAERIQC peptide conjugated to keyhole limpet hemocyanin (KLH), and purified human recombinant caspase-3-truncated His_6_-NMT2 produced as previously described [14]. ImmunoPlus was used to break the immune tolerance for these peptides. Five mice were immunized with each immunogen and screened with test bleeds for reactivity. Hybridomas were produced by the fusion of splenocytes with the murine myeloma non-secreting cell line, PC/NSI/1-AG4-1, and maintained in RPMI 1640 growth medium supplemented with 20% heat-inactivated fetal bovine serum. After positive screening with the immunogen and counter screening for specificity using purified recombinant full length His_6_-NMT1 and His_6_-NMT2 proteins to ensure the absence of cross-reactivity [14], supernatants of 20 NMT1 clones and 26 NMT2 clones were tested for performance in Western blotting and immunohistochemistry on formalin-fixed tissues. Subcloning, expansion, and cryopreservation of 5 well-performing hybridomas were completed for each antigen. Roller bottle cell cultures were used for clonal expansion and cell purification.

### Formalin-fixed paraffin embedded tissue preparation and immunostaining

All human tissues were obtained after review and approval of our local research ethics committee, protocol ETH #25928. Tissue microarrays from participants in the BCIRG 001 clinical trial [16] were obtained. The TMA slides contained formalin-fixed paraffin embedded triplicate core tissue samples (1.0 mm in diameter) from each tumor pretreatment. 5 micron thick TMA slices were deparaffinized, rehydrated, and antigen retrieval performed with 0.05% citraconic anhydride epitope retrieval buffer (details in Supplementary Methods). Overnight primary antibody incubation with NMT1 mAb 6F8D5 (1:100) and the NMT2 mAb 6C5E8 (1:1000) was followed by Dako Envision + HRP labeled secondary antibody for 1 h at room temperature. Slides were then stained with Dako DAB + chromagen, 1% CuSO_4_, and haematolyxlin. Omitting primary antibody served as a negative control in all experiments.

### Immunohistochemistry scoring

Immunohistochemistry was assessed and ordinally scored by two expert breast cancer pathologists blinded to clinical characteristics and outcomes. Given different dynamic experimental ranges of staining intensity between NMT1 and NMT2, both were optimally assessed using respectively three and four ordinal classes. For NMT1, the tumors showing the strongest staining were scored as 2, and tumors showing undetectable staining were scored as 0. A score of 1 indicates a weak-moderate signal. For NMT2, the tumors showing the strongest staining were scored as 3, and the tumors with less than 10% of cells (any intensity) were scored as 0. A score of 1 indicates a weak signal (with > 10% of stained cells) and a score of 2 indicates a moderate signal (with > 10% of stained cells). For each clone, the cytologic pattern and the percentage of cells staining was also evaluated and recorded where applicable.

### Oncoline cell viability assay

PCLX-001, also known as DDD86481, was synthesized as described before [15] and provided in kind by Pacylex Pharmaceuticals Inc. (www.pacylex.com) in a semi-crystalline ·2H_2_O·2HCl salt (M.W. 644.47 g/mol). Cell line viability assay following PCLX-001 addition was measured using the Oncolines™ robotic platform (Netherlands Translational Research Center B.V.). Cells were diluted in the corresponding ATCC recommended medium and dispensed in a 384-well plate at a density of 200–6400 cells per well in 45 µl medium. For each cell line, optimal cell density was used. Plate margins were filled with phosphate-buffered saline and plated cells were incubated in a humidified atmosphere of 5% CO_2_ at 37 °C. After 24 h, 5 µL of diluted PCLX-001 was added and plates were further incubated. At *t* = end, 24 µL of ATPlite 1Step™ (PerkinElmer) solution was added to each well, and subsequently shaken for 2 min. After 10 min of incubation in the dark, the luminescence was recorded on an Envision multimode reader (PerkinElmer). IC_50_ values were calculated by non-linear regression using IDBS XLfit 5. The percentage growth after incubation was calculated as:

$$100\% \, \times \, \left( {{\text{luminescence}}_{{t \, = {\text{ end}}}} /{\text{luminescence}}_{{{\text{untreated}},t = {\text{end}}}} } \right).$$

This was fitted to the ^10^log compound concentration by a 4-parameter logistics curve:

$$\left( \% \right)\;{\text{growth}} = {\text{bottom}} + \, \left( {{\text{top }}{-}{\text{ bottom}}} \right) \, / \, \left( {1 + \, 10^{{\left( {\log IC50 \, {-}{\text{conc}}} \right)*{\text{hill}})}} } \right),$$

where hill is the Hill-coefficient, and bottom and top the asymptotic minimum and maximum cell growth that the compound allows in that assay. LD_50_, was calculated as:

$${\text{luminescence}}_{{t = {\text{end}}}} = \, \raise.5ex\hbox{$\scriptstyle 1$}\kern-.1em/ \kern-.15em\lower.25ex\hbox{$\scriptstyle 2$} \, \times \;{\text{luminescence}}_{t = 0\;h} .$$

GI_50_, was associated with the signal:

$$\left( {\left( {{\text{luminescence}}_{{{\text{untreated}},t = {\text{end}}}} {-}{\text{luminescence}}_{t = 0} } \right) \, /2} \right) \, + {\text{luminescence}}_{t = 0 \, .}$$

### MDA-MB-231 cell line derived murine xenografts

Pharmaron (Beijing, P.R. China) was contracted by Pacylex to perform the xenograft experiments. MDA-MB-231 breast cancer cells (1 × 10^7^) were subcutaneously injected into on the right lower flank of thirty 6–8 week old NODscid mice at Pharmaron laboratory facilities (Beijing, China). Once the mean tumor size reached approximately 125 mm^3^, mice were randomized to receive an oral daily dose of either 35 mg/kg or 50 mg/kg of PCLX-001, or vehicle alone for 21 days. Treatment groups consisted of 10 mice each. Body weight, tumor measurements, and other routine monitoring was performed on all study animals once every other day (once per 2 days) throughout the course of the study. Body weight changes were calculated as:

$${\text{BW change }}\left( \% \right) \, = \, \left( {{\text{BW Day X }}/{\text{BW Day }}0} \right) \, \times { 1}00$$

Tumor measurements were conducted using calipers and tumor volume was estimated as:

$${\text{Tumor volume }} = \, \left( {{\text{length }}\left( {{\text{mm}}} \right) \, \times {\text{ width }}\left( {{\text{mm}}} \right)} \right)^{{2}} /{2}$$

where “length” and “width” were the longest and shortest diameters of a tumor, respectively.

Tumor growth inhibition (TGI) was calculated using the formula:

$$TGI \, = \, \left( {1 - T/C} \right) \, \times \, 100\%$$

where “*T*” and “*C*” was the mean relative volumes (% tumor growth) of the tumors in the treated and the control groups, respectively. All the procedures related to animal handling, care, and treatment were performed according to guidelines approved by the Institutional Animal Care and Use Committee (IACUC) of Pharmaron following the guidance of the Association for Assessment and Accreditation of Laboratory Animal Care (AAALAC).

### Statistical analysis

NMT1 and NMT2 staining intensities were correlated with pathological and clinical characteristics using Spearman correlation coefficients, Pearson’s r, and Chi-square calculated with SAS version 6.12 and SPSS version 26. The level of significance was set at *P* ≤ 0.05 for all analysis. Kaplan-Myer logrank analyses were performed using MedCalc. Analysis of the significance of drug treatments on tumor volume was assessed by two-way ANOVA.

## Results

### Anti-NMT1 and anti-NMT2 mAb validation and confirmation of epitope stability

**Fig. 1:**
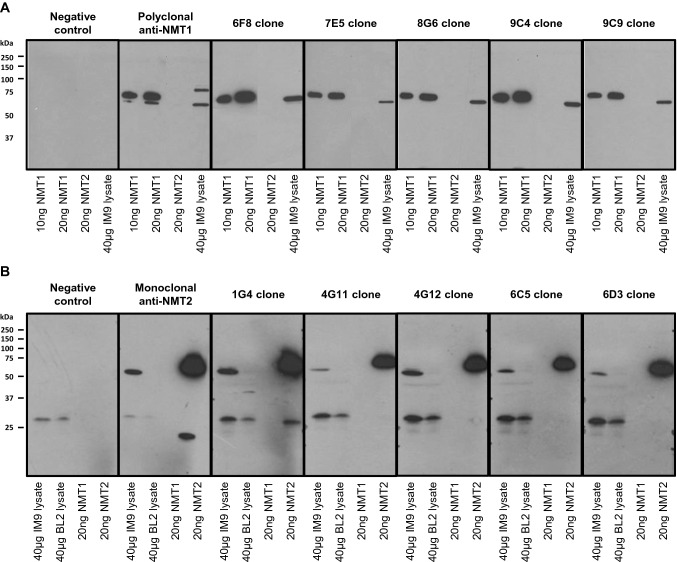
Identification and validation of mouse monoclonal anti-NMT1 and anti-NMT2 antibodies. Western blot of 5 lead NMT1 (**a**) and NMT2 (**b**) mouse monoclonal antibody hybridoma clone supernatants. The negative control was processed without the addition of primary antibody. While both human NMT1 and NMT2 are present in IM9 cell line, the BL2 cell line is NMT2-deficient. Unedited western blots are presented in Supplementary Fig. 1

Mouse monoclonal antibodies raised against human NMT1 and NMT2 (anti-NMT1 mAbs and anti-NMT2 mAbs) were generated as described in the material and methods. Panels of five lead mouse anti-NMT1 and anti-NMT2 mAbs were initially screened for positive NMT1 or NMT2 reactivity and respective absence of cross-reactivity against NMT2 and NMT1 cross-reactivity using both recombinant full length His_6_-NMT1 and His_6_-NMT2 proteins as well as native NMT1 and NMT2 proteins from human IM9 and NMT2-deficient BL2 cell line lysates by immunoblotting (Fig. 1). Antibody panels were then tested for immunohistochemistry performance on formalin-fixed paraffin embedded tissues. The NMT1 mAb 6F8D5 (IgG2b lambda) was selected for further experimentation base upon its high NMT1 affinity, low background staining, reproducible performance, and correspondence with cell line lysate staining using known NMT1 protein abundance on Western blotting.

Similarly, we selected the NMT2 mAb 6C5E8 (IgG1 kappa) for further experimentation based upon its high NMT2 affinity, low background staining, reproducible performance, and correspondence with cell line pellet staining using known NMT2 protein abundance on Western blotting. Epitope stability experiments were also performed using breast tissue samples that had been stored at room temperature in paraffin embedded tissue blocks for 12 years. Indistinguishable NMT1 and NMT2 immunohistochemistry staining results were obtained using both 6F8D5 and 6C5E8 mAbs on these differentially stored samples further validated the use of these mAbs in IHC assays (data not shown) .

### Relationships among NMT protein abundance and clinico-pathologic variables in breast cancer samples

#### NMT1 is variably expressed in breast adenocarcinomas and is associated with proliferation rate

**Fig. 2:**
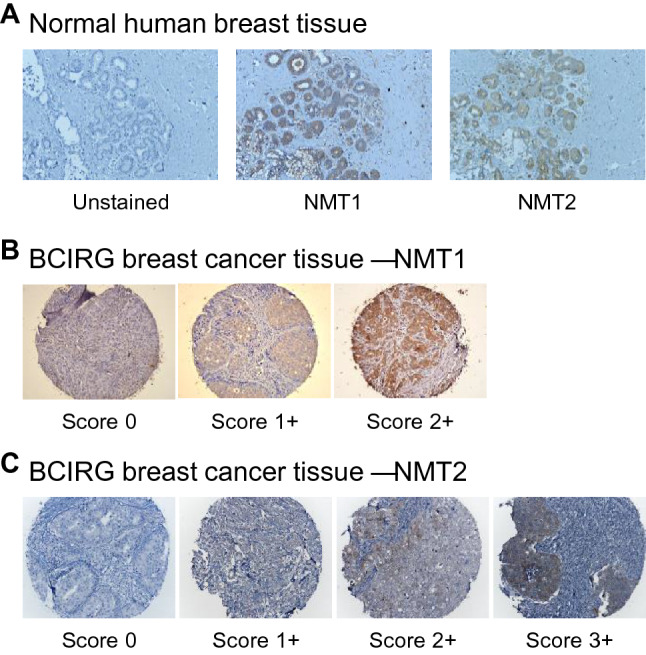
Both NMT1 and NMT2 can readily be detected in normal breast tissue while NMT2 protein is undetectable in a large subset of breast adenocarcinoma tissue samples. **a** Scoring and representative IHC staining for NMT1 and NMT2 protein levels in normal breast epithelial tissue samples. Negative control is tissue sections processed for IHC staining without the addition of primary antibody. **b** Scoring and representative IHC staining for NMT1 and **c** NMT2 protein levels in breast adenocarcinoma tissue samples

To begin our assessment of NMT1 expression in breast carcinoma, we compared the relative expression of NMT1 proteins in normal and cancerous tissue samples using IHC. Normal breast epithelial elements adjacent to tumor on the TMA were universally positive for NMT1 (1–2+) on a 0–2 scale, scored as 0—absent; 1+ weak/moderate staining; 2+ strong staining (Fig. 2a). 602 of 666 primary breast adenocarcinoma tumor samples obtain from participants in the BCIRG 001 clinical trial [16] were found to be NMT1 positive (Table 1 and Fig. 2b). NMT1 was generally homogeneous within each core and concordant across triplicate samples. NMT1 expression was correlated with hormone receptor status, grade, and Ki67 status.

**Table 1:** NMT1 and NMT2 staining intensity on breast adenocarcinoma TMA

Average staining intensity of triplicate cores	Number of tumors			
	0	0.03–1.00	1.03–2.00	2.03–3.00
NMT1 (*n* = 666)	64	280	322	n/a
NMT2 (*n* = 706)	509	142	37	18

#### NMT2 is variably expressed in breast adenocarcinomas and is associated with disease prognosis in a large cohort of breast cancer patients

To begin our assessment of NMT2 expression in breast carcinoma, we compared the relative expression of NMT2 proteins in normal and cancerous tissue samples using IHC. Normal breast epithelial elements adjacent to tumor on the TMA were universally positive for NMT2 (1–2+) on a 0–3 scale, scored as 0 absent; 1+ weak staining; 2+ moderate staining; 3+ strong staining (Fig. 2a). By comparison, 509 of 706 primary breast adenocarcinoma tumor samples obtain from participants in the BCIRG 001 clinical trial [16] were found to be NMT2 negative (Table 1 and Fig. 2c). This suggests that NMT2 loss occurs in the majority of cases, perhaps underlying some new biology linked to the process of carcinogenesis. NMT2 status was generally homogeneous within each core and concordant across triplicate samples.

**Fig. 3:**
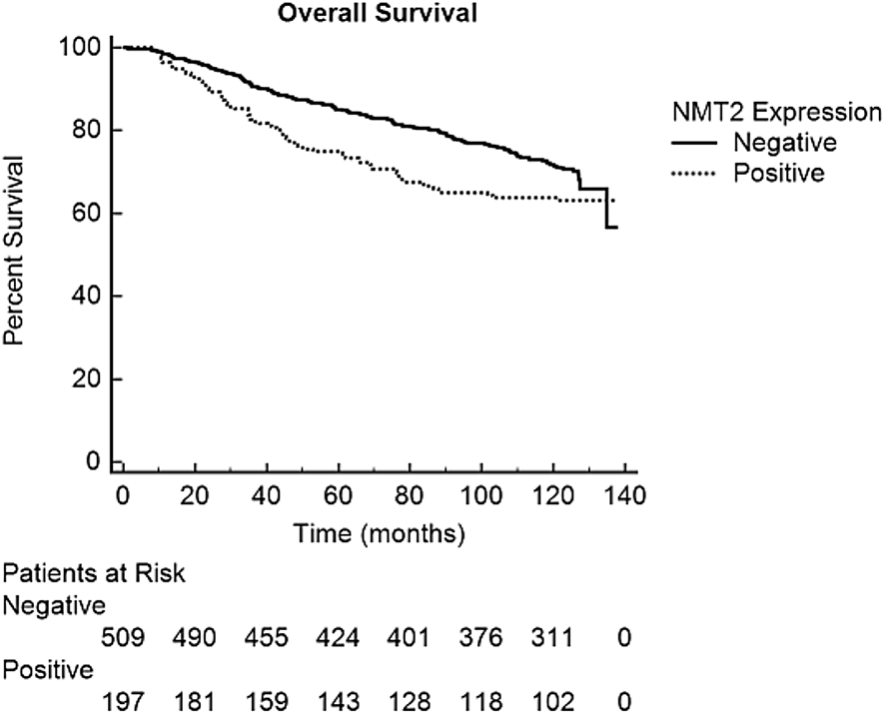
Breast cancer patient overall survival is associated with primary tumor NMT2 status. Kaplan–Meier overall survival analysis based on NMT2 score of 706 breast adenocarcinoma tissue samples stained with 6C5E8 NMT2 mAb. The solid line represents survival outcomes of patients with a negative NMT2 score (*N* = 509) while the dashed line represents survival outcomes of patients with a positive NMT2 score (*N* = 197). (logrank test *P* = 0.029)

To determine if differences in NMT2 protein levels correlated with disease prognosis in these breast cancer patients, we performed Kaplan–Meier overall survival (OS) analysis using the clinical outcomes associated with each breast adenocarcinoma sample. Patients with NMT2 positive tumors were found to have significantly poorer overall survival outcomes (hazard ratio for death 1.36; *P* = 0.029; Fig. 3) and significantly worse biological features including younger age, higher histologic grade, lower hormone receptor expression, higher Ki67, and p53 positivity than patients with NMT2 negative tumors (Table 2). This suggests a new link between the level of NMT2 expression and disease progression. Multivariate analysis incorporating tumor size, tumor grade, axillary node status, and allocated adjuvant chemotherapy regimen failed to show an independent prognostic value for NMT2. This suggests that while NMT2 may relate to breast cancer outcomes, it does so in parallel with other known prognostic factors.

**Table 2:** Statistical relationships among NMT1 (6F8D5) and NMT2 (6C5E8) monoclonal antibody staining and clinicopathologic variables

Clinicopathologic variable	NMT1 relationship	*P*-value	NMT2 relationship	*P*-value
Age of patient	*r* = − 0.058	0.133	*r* = − 0.102	0.007
Estrogen receptor status	*X*^2^ = 16.417	0.000	*X*^2^ = 118.605	0.000
Progesterone receptor status	*X*^2^ = 26.124	0.000	*X*^2^ = 69.561	0.000
Peritumoral lymphovascular invasion	*X*^2^ = 0.490	0.783	*X*^2^ = 5.040	0.169
Overall histologic grade (I,II,II)	*ρ* = + 0.162	0.000	*ρ* = + 0.327	0.000
Mitotic grade	*ρ* = + 0.133	0.001	*ρ* = + 0.376	0.000
Architectural grade	*ρ* = + 0.189	0.000	*ρ* = + 0.120	0.001
Nuclear grade	*ρ* = + 0.089	0.022	*ρ* = + 0.206	0.000
HER2 status	*ρ* = − 0.017	0.659	*ρ* = − 0.052	0.170
Ki67	*r* = + 0.183	0.000	*r* = + 0.492	0.000
p53	*r* = + 0.071	0.069	*r* = + 0.189	0.000
p53 staining intensity	*ρ* = + 0.072	0.114	*ρ* = + 0.207	0.000
Progression free survival	*r* = − 0.001	0.977	*r* = − 0.111	0.003
Overall survival	*r* = − 0.040	0.562	*r* = − 0.271	0.000
NMT2 expression	*ρ* = + 0.146	0.000	na	na

Clinicopathologic relationships with NMT1 and NMT2 expression were analysed with Chi-square test (X^2^), Pearson Correlation (r), or Spearman’s Rank Correlation (*ρ*)

### The pan-NMT inhibitor PCLX-001 kills breast cancer cell lines in vitro

Because IHC experiments detected variability in the expression level of NMT2 in breast adenocarcinoma tissues samples, and NMT2 protein loss correlated with better survival outcomes in breast cancer patients, we sought to determine if inhibiting NMT function in breast cancer lines would reduce cell viability in vitro. Using the Oncolines™ robotic drug testing platform three breast carcinoma (BT-20, DU4475, and Hs 578 T), one breast ductal carcinoma (BT-549), and two breast adenocarcinoma cell lines (AU-565, MCF7) were treated with increasing concentrations (3.16–31,600 nM) of the pan-NMT inhibitor PCLX-001 for 72hrs, which allowed us to calculate IC_50_, GI_50_, and LD_50_ values (Table 3). Several breast cancer cell lines, including Hs 578 T, were markedly sensitive to NMT inhibition with IC_50_ and GI_50_ values in the 100 nM-200 nM and 75–200 nM range, respectively. In contrast, the cell line BT-20 exhibited exceptional resistance to PCLX-001 treatment with IC_50_ and GI_50_ values of > 28,000 nM and > 21,000 nM respectively.

**Table 3:** Breast cancer cell line sensitivity to PCLX-001

Breast cancer cell line	IC_50_ (nM)	GI_50_ (nM)	LD_50_ (nM)
AU-565	193	608	> 10,000
BT-20	28,446	21,323	31,600
BT-549	130	141	> 10,000
DU4475	636	428	1930
Hs 578T	111	77	941
MCF	210	178	> 10,000

IC_50_, GI_50_, and LD_50_ were calculated from cell viability curves (Supplementary Fig. 2 )

### PCLX-001 treatment inhibits tumor growth in a murine MDA-MB-231 breast cancer xenograft model

**Fig. 4:**
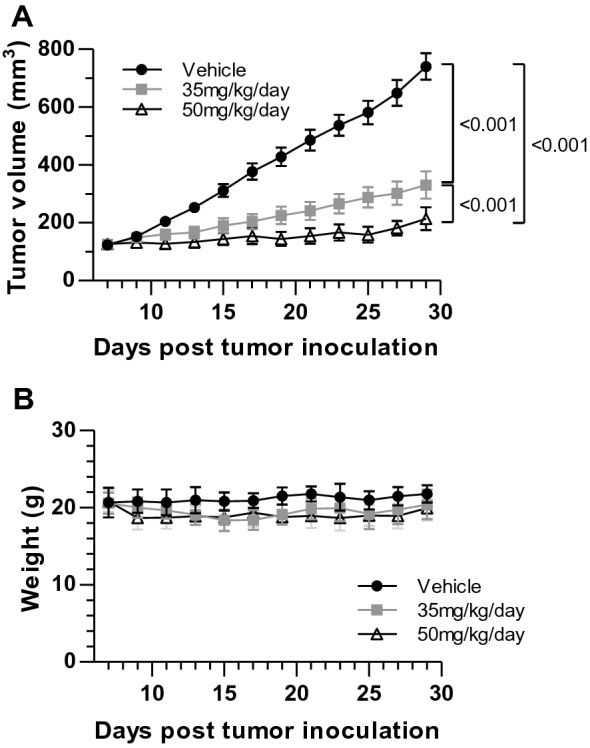
PCLX-001 inhibits breast cancer growth in a murine MDA-MB-231 xenograft model. **a** Tumor volume in mice bearing MDA-MB-231 xenografts over 21 days of oral PCLX-001 administration daily at either 35 mg/kg or 50 mg/kg post tumor inoculation. **b** Average body weight of mice treated with daily 35 mg/kg or 50 mg/kg of PCLX-001 or vehicle alone for 21 days post tumor inoculation

Based on the sensitivity of breast cancer cells to PCLX-001 treatment in vitro, we sought to determine if similar effects could be observed on tumor cell growth using an in vivo breast tumor xenograft model. To this end, a 1 × 10^7^ cell suspension of MDA-MB-231 breast cancer cells were injected into the right flanks of 30 NODscid mice and allowed to propagate until solid tumors of approximately 125 mm^3^ had formed. Tumor bearing mice were then randomized into 3 groups of 10 to receive an oral daily dose of either 35 mg/kg or 50 mg/kg of PCLX-001, or vehicle alone for 21 days. At completion of the experiment, 55.7% tumor growth inhibition (TGI; *P* < 0.001) was observed in mice which received daily 35 mg/kg administration of PCLX-001, and this was increased to 71.8% TGI (*P* < 0.001) when the PCLX-001 dose was increased to 50 mg/kg (Fig. 4a). Moderate weight loss (10.6%) was observed over the course of the experiment in animals which received 35 mg/kg of PCLX-001 (Fig. 4b), with 6 mice requiring a treatment interruption to recover from body weight loss of more than 15% during the second week of treatment. More significant effects were observed in animals which received 50 mg/kg of PCLX-001 daily (Fig. 4b) with treatment interruptions due to weight loss occurring at earlier time points in this treatment regimen, and 6 of 10 animals surviving the 21 day treatment. While further more comprehensive studies are required to determine the optimal therapeutic window for this drug*,* these early proof-of-concept results demonstrate significant in vivo dose-dependent anti-tumor activity for PCLX-001 against solid breast cancer tumors using this xenograft animal model.

## Discussion

NMTs play critical regulatory roles in cell signaling by catalyzing the key modification of cytosolic proteins with the fatty acid myristate directing the resulting fatty acylated proteins to various membranes where key signaling events linked to oncogenesis originate. Despite this key function, our knowledge of the physiological and pathophysiological distributions of NMTs in various tissues is lacking due, in part, to the absence of highly selective monoclonal antibodies against human NMT1 and NMT2 variants. The accurate identification of NMT1 and NMT2 protein levels may be of particular importance in cancer since numerous cancer types were previously demonstrated to exhibit variabilities in NMT expression/protein levels and because of this may be targeted with NMT-directed therapeutics. After generating and validating our lead isotype-specific monoclonal antibodies against NMT1 and NMT2, we identified variations in NMT2 protein levels in malignant breast epithelial tissue using IHC staining. While a very high proportion of breast cancer samples had detectable NMT1 protein levels (602 of 666 tumors), a large proportion (509 of 706 tumors) exhibited very low or undetectable amounts of NMT2 despite normal breast epithelia being ubiquitously positive for NMT2 proteins. These data suggest that NMTs are clearly not always overexpressed in breast cancers and that this conclusion may be cancer specific. This novel finding suggests that loss of *NMT2* expression may occur, variably, in breast carcinogenesis underlying some potentially new biology. Kaplan–Meier survival analysis demonstrated a correlation between NMT2 positive detection and poorer prognosis. Importantly, NMT2 protein level was not an independent prognostic factor, suggesting NMT2 status is related to standard prognostic factors included in our study. With more than 364 different proteins requiring myristoylation for their function in human cells [3], the mechanisms by which NMT2 status relates to individual myristoylated proteins and clinical outcomes are still a matter of speculation, but are under investigation in our laboratory.

Because loss of NMT2 protein in breast cancer favored better patient prognosis, we investigated whether breast cancer cells were susceptible to NMT inhibition using the pan-NMT inhibitor PCLX-001 both in vitro, and in an in vivo animal model. Responses to PCLX-001 were highly variable in the breast cell lines tested with some being markedly sensitive to NMT inhibition and others appearing inherently resistant. Importantly, PCLX-001 also demonstrated a significant dose-dependent inhibitory effect on breast cancer cell growth when administered to mice bearing MDA-MB-231 breast cancer cell line xenografts, validating the therapeutic potential of PCLX-001 in the treatment of solid breast tumors in vivo. Formal studies are underway to evaluate the pharmacokinetics and toxicology of PCLX-001 to facilitate its clinical evaluation in human cancers [12].

Ultimately, as NMT inhibitors move toward clinical trials as anticancer therapeutics, NMT1and NMT2 monoclonal antibodies may prove invaluable for rational selection of patient populations for clinical trials, and potentially provide predictive assays for selection of sensitive patients in clinical practice. As NMT2 expression can be lost during carcinogenesis and carries prognostic value, there may be additional biology, yet to be revealed, that can be exploited to further improve breast cancer patient outcomes.

## Supplementary Information

Below is the link to the electronic supplementary material.

Supplementary Fig. 1 Identification and validation of mouse monoclonal anti-NMT1 and anti-NMT2 antibodies. Western blot of 5 lead NMT1 (A) and NMT2 (B) mouse monoclonal antibody hybridoma clone supernatants. The negative control was processed without the addition of primary antibody. While both human NMT1 and NMT2 are present in IM9 cell line, the BL2 cell line is NMT2-deficient. The molecular weight marker used was Precision Plus Protein All Blue (BioRad). (PPTX 2524 kb)

Supplementary Fig. 2 Breast cancer cell lines exhibit variable sensitivity to the pan-NMT inhibitor PCLX-001 based on NMT expression levels. Cell viability curves of Au-565, BT-20, BT-549, DU4475, Hs 578T and MCF7 breast cancer cell lines treated with 3.16nM – 31,600nM of PCLX-001 for 72hr. (PPTX 305 kb)

Electronic supplementary material 3 (DOCX 13 kb)
