# Supplementary material for: N-myristoyltransferase proteins in breast cancer: prognostic relevance and validation as a new drug target
Source: Breast Cancer Res Treat. 2021 Jan 4;186(1):79–87. doi: 10.1007/s10549-020-06037-y (PMC7940342; doi:10.1007/s10549-020-06037-y)
Supplement: Supplementary file 1 — Supplementary Fig. 1 Identification and validation of mouse monoclonal anti-NMT1 and anti-NMT2 antibodies. Western blot of 5 lead NMT1 (A) and NMT2 (B) mouse monoclonal antibody hybridoma clone supernatants. The negative control was processed without the addition of primary antibody. While both human NMT1 and NMT2 are present in IM9 cell line, the BL2 cell line is NMT2-deficient. The molecular weight marker used was Precision Plus Protein All Blue (BioRad). (PPTX 2524 kb) [file 10549_2020_6037_MOESM1_ESM.pptx]

## Slide 1
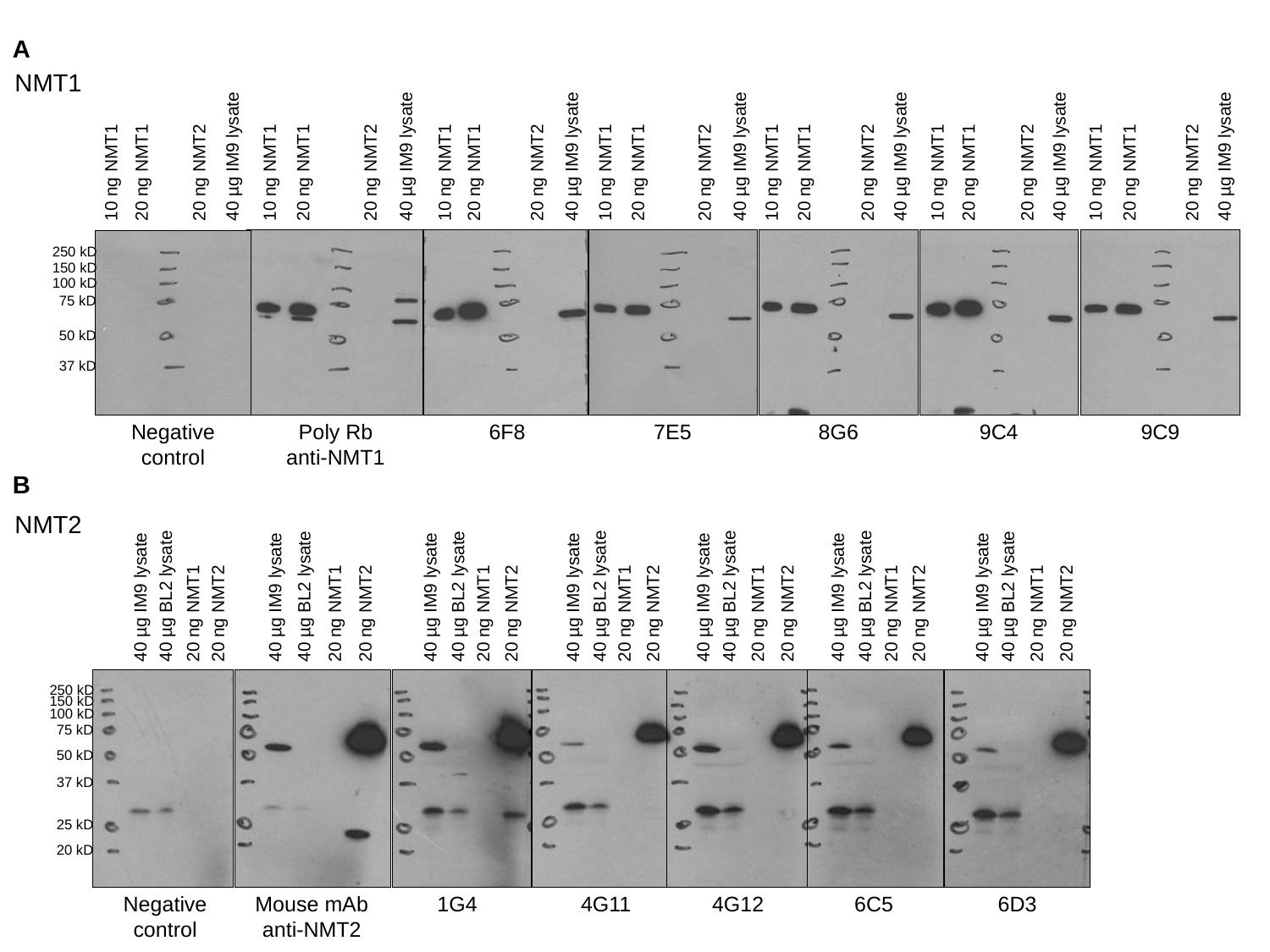

A
NMT1
40 µg IM9 lysate
40 µg IM9 lysate
40 µg IM9 lysate
40 µg IM9 lysate
40 µg IM9 lysate
40 µg IM9 lysate
40 µg IM9 lysate
10 ng NMT1
20 ng NMT1
20 ng NMT2
10 ng NMT1
20 ng NMT1
20 ng NMT2
10 ng NMT1
20 ng NMT1
20 ng NMT2
10 ng NMT1
20 ng NMT1
20 ng NMT2
10 ng NMT1
20 ng NMT1
20 ng NMT2
10 ng NMT1
20 ng NMT1
20 ng NMT2
10 ng NMT1
20 ng NMT1
20 ng NMT2
250 kD
150 kD
100 kD
75 kD
50 kD
37 kD
Negative
control
Poly Rb
anti-NMT1
6F8
7E5
8G6
9C4
9C9
B
NMT2
40 µg BL2 lysate
40 µg BL2 lysate
40 µg BL2 lysate
40 µg BL2 lysate
40 µg BL2 lysate
40 µg BL2 lysate
40 µg BL2 lysate
40 µg IM9 lysate
40 µg IM9 lysate
40 µg IM9 lysate
40 µg IM9 lysate
40 µg IM9 lysate
40 µg IM9 lysate
40 µg IM9 lysate
20 ng NMT1
20 ng NMT2
20 ng NMT1
20 ng NMT2
20 ng NMT1
20 ng NMT2
20 ng NMT1
20 ng NMT2
20 ng NMT1
20 ng NMT2
20 ng NMT1
20 ng NMT2
20 ng NMT1
20 ng NMT2
250 kD
150 kD
100 kD
75 kD
50 kD
37 kD
25 kD
20 kD
Negative
control
Mouse mAb
anti-NMT2
1G4
4G11
4G12
6C5
6D3
