# Supplementary figures and images for: N-myristoyltransferase proteins in breast cancer: prognostic relevance and validation as a new drug target
Source: Breast Cancer Res Treat. 2021 Jan 4;186(1):79–87. doi: 10.1007/s10549-020-06037-y (PMC7940342; doi:10.1007/s10549-020-06037-y)

## Slide 1
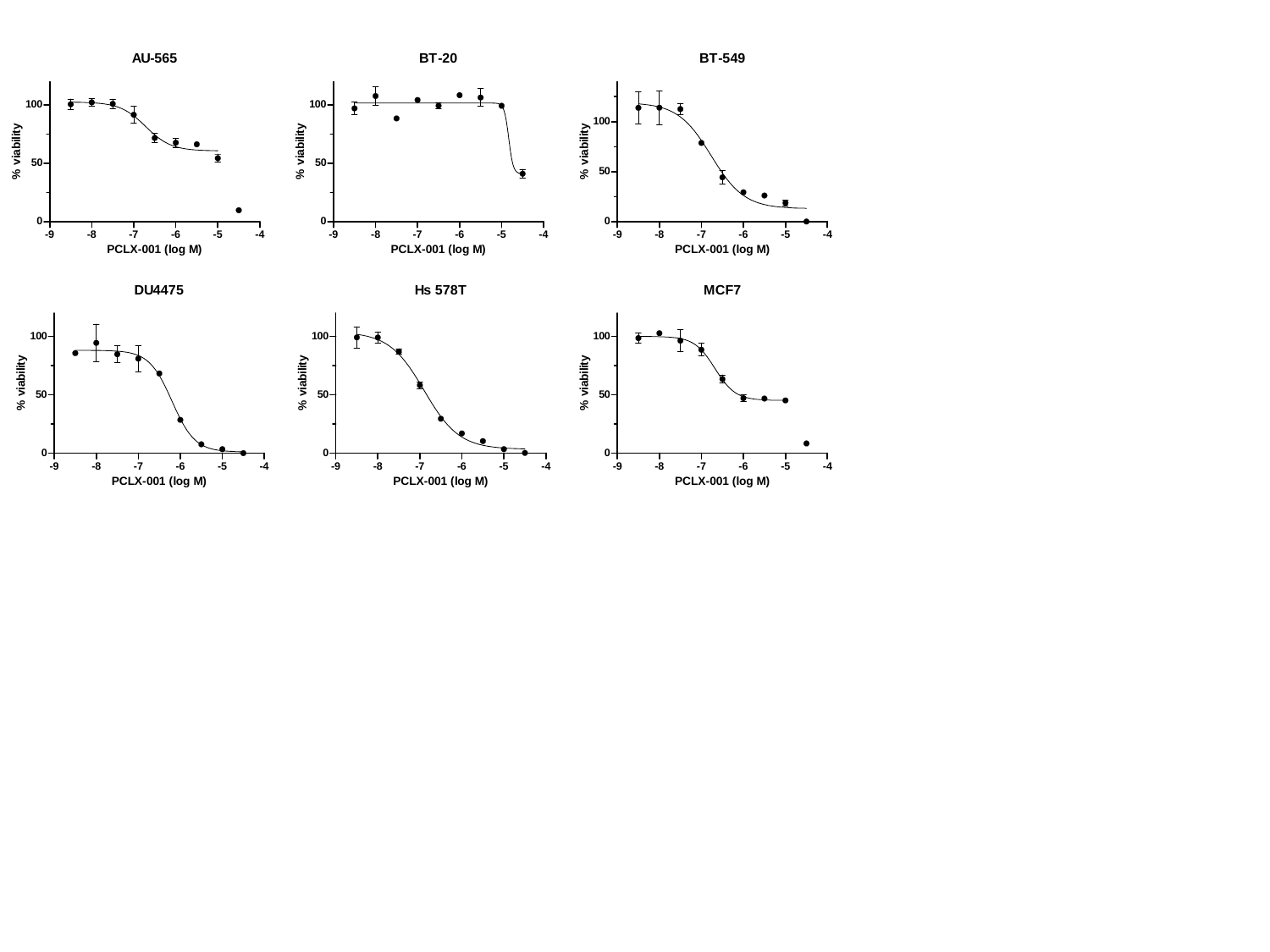

Supplement: Supplementary file 2 — Supplementary Fig. 2 Breast cancer cell lines exhibit variable sensitivity to the pan-NMT inhibitor PCLX-001 based on NMT expression levels. Cell viability curves of Au-565, BT-20, BT-549, DU4475, Hs 578T and MCF7 breast cancer cell lines treated with 3.16nM – 31,600nM of PCLX-001 for 72hr. (PPTX 305 kb) [file 10549_2020_6037_MOESM2_ESM.pptx]
