# Supplementary material for: N-myristoyltransferase proteins in breast cancer: prognostic relevance and validation as a new drug target
Source: Breast Cancer Res Treat. 2021 Jan 4;186(1):79–87. doi: 10.1007/s10549-020-06037-y (PMC7940342; doi:10.1007/s10549-020-06037-y)
Supplement: Supplementary file 3 — Electronic supplementary material 3 (DOCX 13 kb) [file 10549_2020_6037_MOESM3_ESM.docx]

**Supplementary Materials and Methods**

Western blotting

Cells were harvested, washed in cold PBS, and lysed in 0.1% SDS-RIPA buffer (50 mM Tris-HCl pH 8.0, 150 mM NaCl, 1% Igepal CA-630, 0.5% sodium deoxycholate, 2 mM MgCl_2_, 2 mM EDTA with 1x complete protease inhibitor; (Roche Diagnostics) by rocking for 15 min at 4ºC. The lysates were centrifuged at 16,000g for 10 min at 4ºC, and the post-nuclear supernatant was collected. Protein concentrations were determined by BCA assay (Thermo Scientific) according to manufacturer’s instructions. Samples were prepared for electrophoresis by the addition of 5X loading buffer and boiled for 5 min.

Immunohistochemistry

5 micron thick formalin-fixed paraffin embedded TMA slices were deparaffinized through xylene and rehydrated in graded alcohols. Antigen retrieval was performed in a microwave pressure cooker system for 6 minutes at 750 watts in preheated epitope retrieval buffer (0.05% citraconic anhydride (from 98% stock, Aldrich), pH 7.4), after which slides were placed in cold running water for 5 mins and then TBST (TBS with 0.05% Tween 20). Slides were blocked with 0.5% fish gelatin in TBST for 30 minutes and then incubated with primary antibody in Dako antibody diluent (DAKO Corporation, Carpinteria, CA). overnight in a humidified chamber at 4ºC. The NMT1 mAb 6F8D5 was used at a dilution of 1:100 and the NMT2 mAb 6C5E8 was used at a dilution of 1:1000. After rinsing with TBS, endogenous peroxidase was blocked using a solution of 3% H_2_0_2_ in TBS for 15 minutes and then slides were washed twice with TBST for 5 minutes each. Slides were incubated with Dako Envision+ HRP labeled secondary antibody for 1 hour at room temperature in a humidified chamber and then rinsed in TBS once and TBST three times and TBS once more. Dako DAB+ chromagen was added to each slide and developed for 3-5 minutes with microscopy monitoring, rinsed for 10 minutes in running tap water, then soaked in 1% CuSO_4_ for 5 minutes and washed. Slides were placed in haematolyxlin for 10 sec, rinsed with tap water until clear, dipped in warm lithium carbonate 2 minutes, rinsed with running tap water, and dehydrated through graded alcohols and xylene, followed by cover slip addition with DPX mounting media (ThermoFisher).
